# Knowledge and barriers of PrEP delivery among diverse groups of potential PrEP users in Central Uganda

**DOI:** 10.1371/journal.pone.0241399

**Published:** 2020-10-28

**Authors:** Timothy R. Muwonge, Rogers Nsubuga, Charles Brown, Agnes Nakyanzi, Monica Bagaya, Felix Bambia, Elly Katabira, Peter Kyambadde, Jared M. Baeten, Renee Heffron, Connie Celum, Andrew Mujugira, Jessica E. Haberer

**Affiliations:** 1 Infectious Diseases Institute Makerere University, Kampala, Uganda; 2 Department of Medicine, College of Health Sciences, Makerere University, Kampala, Uganda; 3 Most At-Risk Populations Initiative, Kampala, Uganda; 4 STD/AIDS Control Program Ministry of Health, Kampala, Uganda; 5 Department of Global Health, Medicine, and Epidemiology, University of Washington, Seattle, Washington, United States of America; 6 Department of General Internal Medicine, Massachusetts General Hospital Global Health, Boston, Massachusetts, United States of America; University of Ghana College of Health Sciences, GHANA

## Abstract

**Background:**

Scale-up of oral pre-exposure prophylaxis (PrEP) for HIV prevention in Uganda began with serodiscordant couples (SDC) and has expanded to other most at-risk populations (MARPs). We explored knowledge, acceptability, barriers and facilitators of PrEP use among potential PrEP users in four MARPs (SDC; men who have sex with men [MSM]; female sex workers [FSW], and fisher folk).

**Methods:**

We administered quantitative surveys to potential PrEP users in multiple settings in Central Uganda at baseline and approximately 9 months after healthcare worker (HCW) training on PrEP.

**Results:**

The survey was completed by 250 potential PrEP users at baseline and 125 after HCW training; 55 completed both surveys. For these 250 participants, mean age was 28.5 years (SD 6.9), 47% were male and 6% were transgender women, with approximately even distribution across MARPs and recruitment locations (urban, peri-urban, and rural). Most (65%) had not heard about PrEP. After HCW training, 24% of those sampled were aware of PrEP, and the proportion of those who accurately described PrEP as “antiretrovirals to be used before HIV exposure” increased from 54% in the baseline survey to 74% in the second survey (p<0.001). The proportion of participants who reported HCW as a source of PrEP information increased after training (59% vs 91%, p<0.001). In both surveys, nearly all participants indicated they were willing to take PrEP if offered. The most common anticipated barriers to PrEP were stigma, transportation, accessibility, busy schedules, and forgetfulness. Closeness to home was a common facilitator for all participant categories.

**Conclusions:**

Initial awareness of PrEP was low, but PrEP knowledge and interest increased among diverse MARPs after HCW training. Demand creation and HCW training will be critical for increasing PrEP awareness among key populations, with support to overcome barriers to PrEP use. These findings should encourage the acceleration of PrEP rollout in Uganda.

## Introduction

Oral pre-exposure prophylaxis (PrEP) using tenofovir (TDF) and emtricitabine (FTC) as a once-daily pill is highly effective against HIV infection [1, 2]; however, the degree of effectiveness depends greatly on uptake and adherence [3]. The World Health Organization (WHO) recommends the use of PrEP for HIV negative persons at high risk of infection [4], including multiple most-at-risk populations (MARPs) such as serodiscordant couples (SDC), men who have sex with men (MSM), female sex workers (FSW), adolescent girls and young women (AGYW), and fisher folk [4].

PrEP is currently being implemented globally with varying degrees of interest among MARPs. Acceptability of PrEP in the United States, Europe, and Australia has been high among MSM, despite access and cost barriers [5, 6]. Data on PrEP acceptability in sub-Saharan Africa are limited; however, a few studies have shown high acceptability among MSM [7, 8] and variable acceptability and adherence among adolescent girls and young women [9, 10]. Several demonstration projects have indicated the benefits of community engagement in creating demand and providing education and adherence support for PrEP service delivery among potential PrEP users [9].

The Ugandan Ministry of Health (MoH) began implementing a PrEP roll-out among SDC in 2017 due to demonstrated high adherence and effectiveness in preventing HIV infection in this population [11, 12]. Given its dedication to offering HIV prevention, care, and treatment to other MARPs as well, the Ugandan MoH has expanded PrEP services in recent years [10, 13, 14]. Training of health care workers (HCWs) may improve PrEP knowledge and awareness among potential PrEP users resulting in higher uptake [15]. However, data on how to facilitate the implementation in routine clinical practice are needed to guide program implementation in Uganda and other sub-Saharan African settings.

To prepare for this rollout, we surveyed potential PrEP users among four key MARPs in Uganda (SDC, MSM, FSW, and fisher folk) before and after HCW training that followed routine MoH guidelines [13, 16, 17]. The government of Uganda had not yet endorsed PrEP use among adolescent girls and young women at the time of data collection and AGYW were therefore not included in the study. We aimed to characterize potential PrEP users in the selected MARPs, assess their knowledge and interest in PrEP, and identify potential barriers and facilitators to PrEP uptake and adherence.

## Methods

### Subjects and setting

This study was implemented by the Infectious Diseases Institute Kasangati (IDIK)—a research clinic adjacent to Kasangati Health Center IV, which is a public health facility located in central Uganda (HIV prevalence 5.8% [18]). At the time of the study, central Uganda had 504 HIV care and treatment facilities including government health centers, sexually transmitted infection/HIV clinics offering HIV testing and treatment services, serodiscordant couple community-based organizations, and non-governmental organizations. We selected a purposive sample of health centers that provided HIV services and not yet providing PrEP at the time of data collection. We included clinics that would be representative of routinely delivered PrEP service and excluded the six pilot facilities that were providing PrEP at the time of data collection. We used these criteria to select 35 health centers, which were stratified in roughly equal proportions, by urban, peri-urban, and rural areas within the central region of Uganda.

### Sampling

Using convenience sampling for SDC and fisher folk, snowball sampling for FSW and MSM, we aimed to recruit 250 potential PrEP users at baseline. Based on anticipated challenges in relocating the initial survey participants, we planned to recruit 125 potential PrEP users for the post-training survey sample. Because these challenges were substantial, additional participants were recruited using methods as described for the first survey and helped to ensure that we assessed broad perspectives. All participants were selected from urban, peri-urban, and rural areas served by the selected health facilities. Inclusion criteria were age 18 years or older, HIV-negative test results performed according to the national HIV testing algorithm [10, 19]; member of a MARP of interest (SDC, MDM, FSW, or fisher folk); and residence within a 70km radius of IDIK. The only exclusion criterion was unwillingness to provide informed consent to participate in the study.

### Survey administration

Survey questionnaires were developed in English and translated into Luganda (the local language). Content-focused on 1) PrEP knowledge (what is PrEP, PrEP eligibility, expected duration of PrEP, sources of PrEP information), 2) PrEP acceptability (willingness to take PrEP), and 3) anticipated barriers and facilitators to PrEP use. The questions developed for this survey were based on national PrEP guidelines [13]. After authors TRM and JEH agreed on survey content, items were pretested among research staff who were not part of the study team for clarity and relevance. Pre-specified options were given for each question, but participants could also indicate other responses. Trained bilingual research assistants in either English or Luganda (depending on participant preference) conducted surveys.

Baseline surveys were administered between August and December 2017, followed by HCW training in January 2018. A second potential PrEP user survey was administered between May 15 and August 7, 2018, which included 55 participants who had taken part in the baseline survey and an additional 70 participants who were newly enrolled. Both surveys were designed to understand participants’ knowledge of PrEP, PrEP acceptability, and anticipated barriers and facilitators of PrEP use. The second survey was used to estimate the impact of the HCW training, as well as broaden our understanding of potential PrEP users’ perspectives with the newly enrolled participants. We also conducted surveys of healthcare workers before and after the training, which indicated improved knowledge and preparedness to offer PrEP, as described elsewhere [20].

### HCW training

In January 2018, we conducted two-day training workshops for urban, peri-urban, and rural HCW in Kampala, Uganda. Training was focused on PrEP knowledge and service delivery, included didactic sessions and role-plays, and was conducted using Uganda MoH training materials [13, 17]. Training modules included: 1) PrEP basics (what is PrEP, differentiating PrEP from PEP and ART, who needs PrEP, identifying people at substantial risk of HIV infection), 2) PrEP screening and eligibility (PrEP eligibility criteria, screening for substantial HIV risk and PrEP eligibility, PrEP contraindications, acute HIV infection), 3) initial and follow-up visits (visit procedures, national HIV testing guidelines, PrEP counseling), and 4) PrEP stigma, side effects, and seroconversion (creatinine elevation, seroconversion management, strategies to minimize PrEP stigma).

### Statistical analysis

A sample size of 250 was chosen to explore and compare point estimates among the four types of MARPs. We assumed participant responses would average in a mid-range and allowed for two-sided confidence intervals of 20–30%. The follow-up cohort sample size was smaller based on anticipated challenges in re-identifying the same participants of potential PrEP users. Survey responses, including PrEP barriers and facilitators, were summarized using descriptive statistics. We used Chi-square tests and ANOVA correlations to compare categorical variables among all participants at baseline, as well as between those surveyed at baseline and after HCW training. Baseline surveys were used to descriptively compare barriers and facilitators among MARPs and by recruitment location. Data analyses were performed using Stata 14 (StataCorp, College Station, TX).

### Ethics considerations

We obtained ethics approval from the National HIV/AIDS Research Committee (ARC 196), at the Uganda National Council for Science and Technology (SS 4277), and Partners HealthCare/ Massachusetts General Hospital (2017P000482/PHS). The Uganda National Council for Science and Technology institutional review board advised that only verbal consent and no locator information be obtained from MSM to protect their privacy. Thus, MSM could not be re-contacted to participate in the second survey. All the other participants provided written or verbal informed consent.

## Results

### Participant characteristics

**Fig 1:**
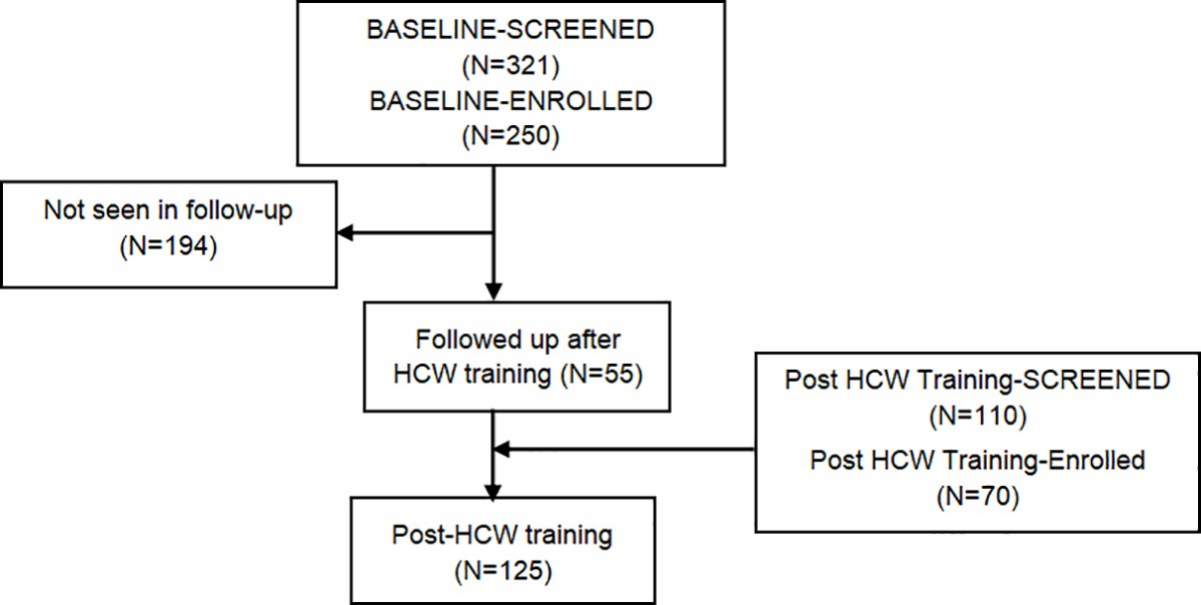
Participant flow.

We screened 321 potential PrEP users and enrolled 250 at baseline. After HCW training, we were able to contact and enroll 55 of the baseline potential PrEP users, and screened 110 new potential PrEP users, of whom 70 enrolled (Fig 1). Those who screened out (71 at baseline and 40 after the HCW training) were not interested in study participation, HIV-positive, or unwilling to be tested for HIV.

The 55 participants who completed surveys both at baseline and after the HCW training are termed the “followed sample”.

Of the 250 potential PrEP users enrolled at baseline, 75 (30%) were MSM, 66 (26%) fisher folk, 56 (22%) FSW and 53 (21%) SDC. The mean age was 28.5 years (SD 6.9) and 92 (37%) were from rural, 85 (34%) from urban and, 73 (29%) from peri-urban settings. Of the 125 potential PrEP users enrolled after HCW training, 31 (25%) were MSM, 31 (25%) fisher folk, 29 (23%) FSW, and 34 (27%) SDC. The mean age was 29.0 years (SD 7.2) and 34 (39%), 29 (34%), and 23 (27%) were from urban, peri-urban, and rural settings, respectively. Demographic characteristics did not differ significantly between the baseline and post-HCW training groups except for a higher mean monthly income in the second survey (US$82.38 versus $99.44; p<0.01). There were significant differences in participant type, marital status, and participant occupation between the followed sample and those newly enrolled for the post-HCW training survey (Table 1).

**Table 1:** Participant characteristics.

	Baseline	Post-HCW training	p value	Followed sample	Newly enrolled	p value
			(baseline vs post-HCW training)			(followed vs newly enrolled)
	(N = 250)	(N = 125)		(N = 55)	(N = 70)	
N (%) or Mean (SD)						
**HIV Risk group**						
SDC	53 (21%)	34 (27%)	0.53	24 (44%)	10 (14%)	<0.001
MSM	75 (30%)	31 (25%)		0 (0%)	31 (44%)*	
FSW	56 (22%)	29 (23%)		12 (22%)	17 (24%)	
Fisher folk	66 (26%)	31 (25%)		19 (34%)	12 (31%)	
Geographic residence*						
Urban	85 (34%)	34 (39%)	0.24	19 (35%)	15 (48%)	0.39
Peri-urban	73 (29%)	29 (34%)		21 (38%)	8 (26%)	
Rural	92 (37%)	23 (27%)		15 (27%)	8 (26%)	
**Age (mean years, (SD))**	28.5 (6.9)	29.0 (7.2)		29.4 (6.3)	28.9 (7.8)	
**Gender**						
Female	118 (47%)	63 (50%)	0.83	32 (58%)	31(44%)	0.05
Male	118 (47%)	56 (45%)		23 (42%)	33 (47%)	
Transgender	14 (6%)	6 (5%)		0 (0%)	6 (9)	
**Marital Status**						
Single	126 (50%)	63 (50%)	0.94	18 (33%)	45 (64%)	0.01
Married (monogamous)	89 (36%)	48 (38%)		30 (55%)	18 (26%)	
Married (polygamous)	12 (5%)	5 (4%)		2 (4%)	3 (4%)	
Separated	20 (8%)	7 (6%)		4 (7%)	3 (4%)	
Divorced	2 (1%)	1 (1%)		0 (0%)	1(1%)	
Widowed	1 (0%)	1 (1%)		1 (2%)	0 (0%)	
Education (mean years [SD])**	9.1 (4.4)	8.6 (3.9)	0.26	7.5 (3.2)	9.5(4.1)	0.06
**Participant’s occupation**						
Professional	25 (10%)	14 (11%)	0.71	0 (0%)	14 (20%)	0.03
Laborer/semi-skilled	52 (21%)	30 (24%)		15 (27%)	15 (21%)	
Trade/sales	76 (30%)	33 (26%)		17 (31%)	16 (23%)	
Farming/animal raising	13 (5%)	5 (4%)		1 (2%)	4 (6%)	
Housewife	10 (4%)	8 (6%)		7 (13%)	1 (1%)	
Student	13 (5%)	3 (2%)		1 (2%)	2 (3%)	
Other	61 (24%)	32 (26%)		14 (25%)	18 (26%)	
** Monthly income (US$)**	$82.38	$99.44	<0.001	$92.2	$100.80	0.06

* MSM could not be included in the followed sample because privacy concerns prevented the collection of locator information as advised by the institutional review board.

** Complete years of education were considered to be the number of years taken to move from one grade to another without counting repeat years.

### PrEP knowledge and acceptability

As shown in Table 2, the number of people who had heard about PrEP significantly increased from 88 (35%) in the baseline survey to 95 (76%) after the HCW training (p<0.001). At baseline, 49 participants (56%) were aware that PrEP refers to antiretrovirals used before potential exposure to HIV, 25 (28%) described it as post-exposure prophylaxis, and 12 (13%) described it as a pill to treat HIV. After HCW PrEP training, 79 (83%) of MARPs accurately described PrEP as antiretrovirals used before exposure to HIV, 12 (13%) described it as post-exposure prophylaxis, and 14 (15%) described it as a pill to treat HIV (p<0.001).

**Table 2:** PrEP knowledge.

	Baseline	Post-HCW Training	p-value	Followed sample	Newly enrolled	p-value
			(baseline vs post-HCW training)			(followed vs newly enrolled)
**Have you ever heard of PrEP?**	N = 250	N = 125		N = 55	N = 70	
** **Yes	88 (35%)	95 (76%)	<0.001	51 (93%)	44 (63%)	<0.001
** **No	162 (65%)	30 (24%)		4 (7%)	26 (37%)	
**Among those who had heard of PrEP**						
Describe PrEP*	N = 88	N = 95		N = 51	N = 44	
** **Pill to treat HIV infection	12 (14%)	14 (15%)	<0.001	6 (12%)	8 (18%)	0.103
** **Pill taken after potential exposure to HIV	25 (28%)	12 (13%)		9 (18%)	3 (7%)	
** **Use of ARV drugs to prevent HIV before exposure	49 (56%)	79 (83%)		42 (82%)	37 (84%)	
** **Other	4(5%)	1 (1%)		0 (0%)	1 (2%)	
Who told you about PrEP?*	N = 88	N = 95		N = 51	N = 44	
** **Health care worker	57 (65%)	90 (95%)	<0.001	50 (98%)	40 (91%)	0.41
** **Media	19 (22%)	3 (3%)		1 (2%)	2 (5%)	
** **Friend	18 (20%)	5 (5%)		2 (4%)	3 (7%)	
** **Other	3 (3%)	1(1%)		0 (0%)	1 (2%)	
Who can benefit from PrEP?*	N = 88	N = 95		N = 51	N = 44	
** **SDC	19 (22%)	32 (34%)	0.16	19 (37%)	13 (30%)	0.003
** **MSM	38 (43%)	26 (27%)		7 (14%)	19 (43%)	
** **FSW	45 (51%)	53 (56%)		22 (43%)	31 (71%)	
** **Fisher folk	20 (23%)	23 (24%)		16 (31%)	7 (16%)	
Others**						
** **HIV-negative persons	13 (15%)	13 (14%)		10 (20%)	3 (7%)	
** **HIV-positive persons	3 (3%)	0 (0%)		0 (0%)	0 (0%)	
** **MARPs	9 (10%)	11 (12%)		5 (10%)	6 (14%)	
** **Raped	7 (8%)	2 (2%)		0 (0%)	2 (5%)	
** **Alcohol users	2 (2%)	3 (3%)		1 (2%)	2 (5%)	
** **Youth	2 (2%)	2 (2%)		1 (2%)	1 (2%)	
** **Breastfeeding mothers	1 (1%)	0 (0%)		0 (0%)	0 (0%)	
** **Don’t know	2 (2%)	9 (9%)		3 (6%)	6 (14%)	
**How long should someone use PrEP?**	N = 88	N = 93		N = 50	N = 43	
** **Lifelong	32 (34%)	23 (25%)	0.17	17 (34%)	6 (14%)	0.36
** **During periods of HIV risk	28 (31%)	34 (37%)		16 (32%)	18 (42%)	
** **A month or less	9 (10%)	6 (6%)		2 (4%)	4 (9%)	
** **3–6 months	4 (4%)	2 (2%)		1 (2%)	1 (2%)	
** **1 year	5 (6%)	2 (2%)		1 (2%)	1 (2%)	
** **2 years	0 (0%)	1 (1%)		0 (0%)	1 (2%)	
** **Don’t know	13 (14%)	25 (27%)		13 (26%)	12 (28%)	

* These questions had multiple responses.

** These were other responses given by the participants.

When participants were asked about, who told them about PrEP, HCW and the media were the most common sources of information cited. The proportion citing HCW increased from 57 (65%) before the training to 90 (95%) after the training (p<0.001). In contrast, there was a decrease in proportion citing the media (19 [22%] vs 3 [3%]) and friends (18 [20%] vs 5 [5%]) before and after HCW training. In the baseline and second surveys, one-third of participants thought that PrEP should be used for a lifetime.

As shown in Table 3, the majority of the potential PrEP users at baseline had never heard about PrEP; however, the highest proportion described PrEP as an ARV drug to prevent HIV acquisition. HCW were reported to be a common source of PrEP information for MSM (26 [72%]) and FSW (17 [68%]) but less than half of fisher folk (7 [47%]). Most, but not all, people within three categories of MARPs recognized themselves as benefitting from PrEP: MSM (26 [72%]), FSW (19 [76%]) and SDC (7 [58%]). PrEP awareness was not common at baseline among potential PrEP users irrespective of the recruitment location. Most (42 [61%]) of the potential PrEP users in urban and peri-urban areas had a more accurate description of PrEP; however, more than half in the rural region described PrEP as PEP. HCW (57 [65%]) were reported to be the common source of PrEP information by participants from different locations.

**Table 3:** Baseline PrEP knowledge by MARP group and recruitment locations.

	MARPs				Recruitment location		
	MSM	FSW	Fisher folk	SDC	Urban	Peri-urban	Rural
	(n = 75)	(n = 56)	(n = 66)	(n = 53)	(n = 85)	(n = 73)	(n = 92)
**Heard of PrEP**							
** **Yes	36 (48%)	25 (45%)	15 (23%)	12 (23%)	43 (51%)	25 (34%)	20 (22%)
** **No	39 (52%)	31 (55%)	51 (77%)	41 (77%)	42 (49%)	48 (66%)	72 (78%)
Describe PrEP*	N = 36	N = 25	N = 15	N = 12	N = 43	N = 25	N = 20
** **Pill to treat HIV infection	2 (6%)	3 (12%)	2 (13%)	5 (42%)	4 (9%)	6 (24%)	2 (10%)
** **Pill taken after potential exposure to HIV	12 (33%)	7 (28%)	4 (27%)	2 (17%)	8 (19%)	6 (24%)	11 (55%)
** **Use of ARV drugs to prevent HIV acquisition before exposure	21 (58%)	16 (64%)	8 (53%)	4 (33%)	30 (70%)	12 (48%)	7 (35%)
** **Other	1 (3%)	1 (4%)	1 (7%)	1 (8%)	2 (5%)	1 (4%)	1 (5%)
Who told you about PrEP*	**N = 36**	**N = 25**	**N = 15**	**N = 12**	**N = 43**	**N = 25**	**N = 20**
** **Health care worker	26 (72%)	17 (68%)	7 (47%)	7 (58%)	34 (79%)	11 (44%)	12 (60%)
** **Media	8 (22%)	2 (8%)	7 (47%)	2 (17%)	6 (14%)	10 (40%)	3 (15%)
** **Friend	6 (17%)	7 (28%)	2 (13%)	3 (25%)	5 (12%)	6 (24%)	7 (35%)
** **Other	2 (6%)	0 (0%)	0 (0%)	1 (8%)	2 (5%)	1 (4%)	0 (0%)
Who can benefit from PrEP?*	N = 36	N = 25	N = 15	N = 12	N = 43	N = 25	N = 20
** **SDC	6 (17%)	3 (12%)	3 (20%)	7 (58%)	11 (26%)	4 (16%)	4 (20%)
** **MSM	26 (72%)	9 (36%)	2 (13%)	1 (8%)	23 (53%)	6 (24%)	9 (45%)
** **FSW	23 (64%)	19 (76%)	3 (20%)	0 (0%)	26 (60%)	7 (28%)	12 (60%)
** **Fisher folk	11 (31%)	7 (28%)	2 (13%)	0 (0%)	10 (23%)	6 (24%)	4 (20%)
Others							
** **HIV-negative persons	3 (8%)	2 (8%)	5 (33%)	3 (25%)	6 (14%)	4 (16%)	3 (15%)
** **HIV-positive persons	1 (3%)	0 (0%)	2 (13%)	0 (0%)	1 (2%)	2 (8%)	0 (0%)
** **MARPs	6 (17%)	3 (12%)	0 (0%)	0 (0%)	3 (7%)	3 (12%)	3 (15%)
** **Raped persons	4 (11%)	3 (12%)	0 (0%)	0 (0%)	2 (5%)	2 (8%)	3 (15%)
** **Alcohol users	2 (6%)	0 (0%)	0 (0%)	0 (0%)	1 (2%)	0 (0%)	1 (5%)
** **Youth	2 (6%)	0 (0%)	0 (0%)	0 (0%)	1 (2%)	0 (0%)	1 (5%)
** **Breastfeeding mothers	0 (0%)	0 (0%)	0 (0%)	1 (8%)	0 (0%)	1 (4%)	0 (0%)
** **Don’t know	1 (3%)	0 (0%)	0 (0%)	0 (0%)	1 (2%)	1 (4%)	0 (0%)
**How long should someone use PrEP?**	N = 36	N = 25	N = 15	N = 12	N = 43	N = 25	N = 20
** **Lifelong	11 (31%)	7 (28%)	7 (47%)	6 (50%)	11 (26%)	15 (60%)	5 (25%)
** **During periods of risk for HIV acquisition	13 (36%)	13 (52%)	2 (13%)	0 (0%)	12 (28%)	7 (28%)	9 (45%)
** **A month or less	5 (14%)	1 (4%)	2 (13%)	1 (8%)	6 (14%)	1 (4%)	2 (10%)
** **3–6 months	2 (6%)	1 (4%)	0 (0%)	1 (8%)	4 (9%)	0 (0%)	0 (0%)
** **1 year	0 (0%)	0 (0%)	2 (13%)	1 (8%)	3 (7%)	0 (0%)	0 (0%)
** **Don’t know	5 (14%)	3 (12%)	2 (13%)	3 (25%)	7 (16%)	2 (8%)	4 (20%)

* These questions had multiple responses.

### Acceptability of PrEP

As shown in Table 4, a high proportion of potential PrEP users were willing to use PrEP to prevent HIV acquisition (232 [93%] at baseline and 107 [85%] in the second survey, p = 0.07). The most common reasons for lack of interest in PrEP were no perceived risk of HIV infection and being satisfied with other HIV prevention tools, such as condoms.

**Table 4:** Acceptability of PrEP.

	Baseline	Post-HCW Training	p-value	Followed samples	Newly enrolled	p-value
			(baseline vs post-HCW training)			(followed vs newly enrolled)
**In case PrEP was made available, would you be willing to use PrEP to prevent HIV infection?**	N = 250	N = 125		N = 55	N = 70	
** **Yes	232 (93%)	107 (85%)	0.07	51 (93%)	56 (80%)	0.10
** **No	15 (6%)	16 (13%)		4 (7%)	12 (17%)	
** **Maybe	3 (1%)	2 (2%)		0 (0%)	2 (3%)	
If no or maybe above, why?*	N = 18	N = 18		N = 4	N = 14	
** **I need more information	3 (17%)	1 (6%)	0.24	0 (0%)	1 (7%)	0.20
** **Not perceived to be at risk	4 (22%)	5 (28%)		3 (75%)	2 (14%)	
** **Happy with other prevention tools like condoms	4 (22%)	5 (28%)		0 (0%)	5 (36%)	
** **I would not want to be seen taking PrEP	2 (11%)	2 (11%)		1 (25%)	1 (7%)	
** **Pill burden	5 (28%)	7 (39%)		1 (25%)	6 (43%)	
** **Side effects	0 (0%)	3 (17%)		0 (0%)	3 (21%)	

* This question had multiple responses.

As shown in Table 5, almost all the MARPs from the different recruitment locations would accept PrEP if it were made available. Across the MARPs who were unwilling to take PrEP, reasons reported included no perceived risk, happy to use other prevention methods, and pill burden. Across the different recruitment areas, pill burden and no perceived risk were most commonly reported among potential PrEP users in the rural region, while preference of other prevention methods was most common among those in the urban region.

**Table 5:** Acceptability of PrEP by MARP group and recruitment locations.

	MARPs				Recruitment location		
	MSM	FSW	Fisher folk	SDC	Urban	Peri-urban	Rural
	(n = 75)	(n = 56)	(n = 66)	(n = 53)	(n = 85)	(n = 73)	(n = 92)
**If PrEP were available, would you be willing to use PrEP to prevent HIV infection?**							
** **Yes	72 (96%)	48 (86%)	62 (94%)	50 (94%)	79 (93%)	68 (93%)	85 (92%)
** **No	3 (4%)	8 (14%)	2 (3%)	2 (4%)	5 (6%)	4 (6%)	6 (7&)
** **Maybe	0 (0%)	0 (0%)	2 (3%)	1 (2%)	1 (1%)	1 (1%)	1 (1%)
If no or maybe above, why?*	N = 3	N = 8	N = 4	N = 3	N = 6	N = 5	N = 7
** **I need more information	1 (33%)	0 (0%)	1 (25%)	1 (33%)	1 (17%)	1 (20%)	1 (14%)
** **Not perceived to be at risk	0 (0%)	0 (0%)	2 (50%)	2 (67%)	1 (17%)	1 (20%)	2 (29%)
** **Happy with other prevention tools like condoms	1 (33%)	3 (38%)	0 (0%)	0 (0%)	2 (33%)	1 (20%)	1 (14%)
** **I would not want to be seen taking PrEP	0 (0%)	2 (25%)	0 (0%)	0 (0%)	1 (17%)	1 (20%)	0 (0%)
** **Pill burden	1 (33%)	3 (38%)	1 (25%)	0 (0%)	1 (17%)	1 (20%)	3 (43%)
** **Side effects	0 (0%)	0 (0%)	0 (0%)	0 (0%)	0 (0%)	0 (0%)	0 (0%)

* This question had multiple responses.

### Barriers and facilitators of PrEP access

As shown in Table 6, several barriers to access PrEP services were reported by participants. In the baseline survey, self-perceived risk of HIV acquisition was high among 136 (53%), moderate in 34 (14%), low in 62 (25%), and none in 11 (4%) and similar in the second survey. Transportation, accessibility of health facilities, and stigma were commonly indicated as barriers. Participant-related factors such as having a busy schedule, forgetting, and alcohol use were also frequently reported hindrances to using PrEP services. Very few participants had concerns about HCW attitudes, PrEP side effects, or their perceived lack of need for PrEP. Notable differences in reported barriers between the baseline and post-HCW training surveys, included forgetting (22 [9%] vs 29 [23%]), accessibility (29 [12%] vs 26 [21%]), cost of the drug (25 [10%] vs 4 [3%]), transport (49 [20%] vs 16 [13%]), and stigma (49 [20%] vs 16 [13%]). However, some barriers were significant at baseline and after HCW training; forgetting (p<0.001), accessibility to health facilities (p = 0.018), drug cost (p = 0.02), alcohol use (p = 0.025) and knowledge gap (p = 0.009).

**Table 6:** Factors potentially influencing PrEP use.

	Baseline	Post-HCW Training	p-value	Followed samples	Newly enrolled	p- value
			(baseline vs post-HCW training)			(followed vs newly enrolled)
	(N = 250)	(N = 125)		(N = 55)	(N = 70)	
**Self-perceived risk of getting HIV**						
** **High	136 (53%)	64 (51%)	0.07	29 (53%)	35 (50%)	0.36
** **Moderate	34 (14%)	8 (6%)		1 (2%)	7 (10%)	
** **Low	62 (25%)	45 (36%)		20 (36%)	25 (36%)	
** **No risk	11 (4%)	5 (4%)		3 (5%)	2 (3%)	
** **Don’t Know	11 (4%)	3 (2%)		2 (4%)	1 (1%)	
Possible barriers to PrEP intake*						
** **Transport	49 (20%)	16 (13%)	<0.001	10 (18%)	6 (9%)	0.23
** **Forgetting	22 (9%)	29 (23%)*		12 (22%)	17 (24%)	
** **Busy schedule	40 (16%)	20 (16%)		7 (13%)	13 (19%)	
** **Accessibility	29 (12%)	26 (21%)*		10 (18%)	16 (23%)	
** **Drug cost	25 (10%)	4 (3%)*		4 (7%)	0 (0%)*	
** **Stigma	49 (20%)	16 (13%)		3 (5%)	13 (19%)*	
** **Alcohol use	11 (4%)	13 (10%)*		6 (11%)	7 (10%)	
** **Pill burden	8 (3%)	6 (5%)		3 (5%)	5 (4%)	
** **Not sure I need it (no perceived risk)	6 (2%)	3 (2%)		2 (4%)	1 (1%)	
** **Drug stock out	17 (7%)	4 (3%)		1 (2%)	3 (4%)	
** **Side effects	4 (2%)	5 (4%)		2 (4%)	3 (4%)	
** **Knowledge gap	1 (0%)	5 (4%)*		2 (4%)	3 (4%)*	
** **HCW attitude	5 (2%)	0 (0%)		0 (0%)	0 (0%)	
Preferred facility for picking PrEP*						
** **District hospital	68 (27%)	27 (22%)	0.06	14 (25%)	13 (19%)	0.28
** **Health center	149 (60%)	65 (52%)		33 (60%)	32 (46%)	
** **Private clinic	23 (9%)	14 (11%)		3 (5%)	11 (16%)	
** **VCT center	17 (7%)	6 (5%)		3 (5%)	3 (4%)	
** **MARPs clinic	38 (15%)	17 (14%)		3 (5%)	14 (20%)	
** **Other	4 (2%)	4 (3%)		2 (4%)	2 (3%)	
Reason why the facility is preferred*						
** **Faster service	38 (15%)	22 (18%)	0.30	6 (11%)	16 (23%)	0.08
** **Less stigma	42 (17%)	28 (22%)		8 (15%)	20 (29%)	
** **Privacy	35 (14%)	17 (14%)		4 (7%)	13 (19%)	
** **Closer to my home	118 (47%)	53 (42%)		31 (56%)	22 (31%)	
** **Accessibility**	6 (2%)	3 (2%)		1 (2%)	2 (3%)	
** **Availability	4 (2%)	2 (2%)		0 (0%)	2 (3%)	
** **Convenience	7 (3%)	4 (3%)		0 (0%)	4 (6%)	
** **Free services	28 (11%)	8 (6%)		6 (11%)	2 (3%)	
** **HCW care/experience	17 (7%)	8 (6%)		2 (4%)	6 (9%)	
** **Reliability/trust	3 (1%)	0 (0%)		0 (0%)	0 (0%)	

** This question referred to preference and comfort of getting to the facility.

The main facilitator reported for using PrEP was the closeness of the PrEP-dispensing facility to home: 118 (47%) and 53 (42%) at baseline and follow-up surveys, respectively. The highest portion of the participants preferred to obtain PrEP from health centers (149 [60%] and 65 [52%], respectively), followed by a district hospital (68 [27%] and 27 [22%]) and a clinic for MARPs (38 [15%] and 17 [14%]). The most common reasons for these preferences included proximity of the facility to their home (118 [47%] and 53 [42%], respectively), less stigma (42 [17%] and 28 [22%]), faster service (38 [15%] and 22 [18%]), and privacy (35 [14%] and 17 [14%]).

Tables 7 and 8 present the most commonly cited barriers and facilitators for each MARP and by recruitment location. Stigma, accessibility, busy schedules, transportation, and drug cost were common; however, the percent endorsing each barrier varied to some degree. Closeness to home was the most common facilitator for all groups and regions. Speed and cost of services and privacy were also commonly noted at varying levels.

**Table 7:** Barriers and facilitators to PrEP access, by MARP group at baseline.

MSM (n = 75)	FSW (n = 56)	Fisher folk (n = 66)	SDC (n = 53)
**Barriers**			
Stigma (41%)	Accessibility (18%)	Busy schedule (24%)	Transportation (26%)
Transportation (15%)	Stigma (16%)	Transportation (23%)	Busy schedule (24%)
Accessibility (15%)	Transportation (16%)	Drug cost (21%)	Stigma (8%)
Busy schedule (13%)	Pill burden (7%)	Forgetting (15%)	Forgetting (8%)
Drug stock out (8%)	Drug cost (5%)	Accessibility (11%)	Drug stock out (8%)
**Facilitators**			
Closeness to home (37%)	Closeness to home (38%)	Closeness to home (58%)	Closeness to home (58%)
Less stigma (35%)	Free services (20%)	Faster services (17%)	Faster services (19%)
Privacy (19%)	Less stigma (16%)	Privacy (12%)	Free services (15%)
HCW care and experience (15%)	Privacy (14%)	Free services (6%)	Less stigma (9%)
Faster services (13%)	Faster services (13%)	HCW care and experience (5%)	Privacy (9%)

**Table 8:** Barriers and facilitators to PrEP access, by recruitment location at baseline.

Urban (n = 85)	Peri-urban (n = 73)	Rural (n = 92)
**Barriers**		
Transportation (24%)	Stigma (19%)	Stigma (18%)
Stigma (21%)	Transportation (19%)	Busy schedule (18%)
Busy schedule (12%)	Busy schedule (18%)	Transportation (16%)
** **Drug cost (12%)	Drug cost (14%)	Accessibility (16%)
Drug stock out (12%)	Accessibility (9%)	Forgetting (10%)
**Facilitators**		
Closeness to home (33%)	Closeness to home (47%)	Closeness to home (61%)
Less stigma (22%)	Faster Service (18%)	Faster services (15%)
Privacy (18%)	Less stigma (15%)	Less stigma (13%)
HCW care and experience (14%)	Free services (15%)	Privacy (12%)
Faster services (13%)	Privacy (12%)	Free services (8%)

## Discussion

In this study, we surveyed four priority MARPs (serodiscordant couples, men who have sex with men, female sex workers, and fisher folk) in central Uganda before and after HCW-training about PrEP services. We found a high willingness to take PrEP among almost all potential PrEP users. The HCW training appeared associated with increased PrEP knowledge and view of HCW as a source of information on PrEP. Transportation and accessibility of health facilities, stigma, busy schedule, forgetting, and alcohol use were commonly indicated as anticipated barriers to PrEP use, while the most common facilitator was the closeness of the PrEP dispensing facility to home. The findings are encouraging for the roll-out of PrEP in Uganda and highlight potential areas for improvement in both HCW training and the design for PrEP services.

Findings of this study indicate high PrEP acceptability among SDC, MSM, FSWs, and fisher folk, which is consistent with key and priority populations in the United States, Europe, Australia, and Kenya [21, 22]. Currently, for geographic areas with high incidence (i.e., >3%), the UNAIDS global target for PrEP rollout by 2025 has been set up to 80% for MSM and FSW and up to 50% for adults above 25 years with multiple sexual partners [23]. With the increase in PrEP acceptability, the Uganda MoH has targeted over 30,000 MARPs to be offered PrEP in 2020–2021 [24]. However, this effort requires strong infrastructure and community preparedness.

Access and cost are key known barriers to PrEP in the United States, Europe, and Australia [5, 6]. Additionally, UNAIDS has indicated that having PrEP in a select group of centers is a barrier to PrEP service delivery due to the long distances to these facilities and attendant transport costs [25, 26]. The participants in our study echoed these concerns. To help overcome such barriers, the Uganda MoH is increasing the number of sites providing PrEP to address the lack of transportation as well as easier and faster access to PrEP from a health center near one’s vicinity [24]. PrEP services are offered free of charge to mitigate the fear of high cost. Pill burden and forgetting were anticipated barriers to PrEP use among this population, as reported in prior studies [27]. We observed that the HCW training increased the coverage of potential PrEP users who received information about PrEP. HCWs are key to reaching MARP communities with PrEP information and services. Training, including practical sessions and role-plays, helps ensure that HCWs are taught communication and counseling skills and can ably address PrEP barriers [28].

The Uganda MoH started the national PrEP roll out in 2017 at six pilot sites [29]. The program has since expanded impressively from 12 to 73 facilities in 2018 and 2019, respectively [24] during which time, the number of clients initiated on PrEP increased substantially to more than 16,000 by September 2019 and implementing districts from 28 to 47. In the 2020 country operating plan, the target number of individuals to be initiated on PrEP is 30,000 persons at 142 facilities out of 1,860 health care facilities providing HIV care services which will require additional expansion of services [24].

This study has a number of important limitations. Although we recruited from a diverse set of clinics, the participants were identified by convenience sampling and may not be representative of each overall population. Additionally, the effects of the HCW training may not be causal and the follow-up survey involved both new and followed participants. Detailed information about HCW training is presented separately in a conference abstract [20]. Due to confidentiality concerns for MARPs, particularly MSM given punitive legal consequences in the country, we were not able to collect locator information to include them in the followed-up sample. That said, combining the two participant groups seemed reasonable, especially as few differences were seen in most responses except as noted. As for strengths, this study is one of the first to explore facilitators and barriers for PrEP use in MARPs in Uganda and provides much-needed guidance for the PrEP rollout in these vulnerable populations. Lessons learned from this study should be considered for both PrEP uptake and persistence during periods of HIV risk.

## Conclusions

Our study shows that people in highly stigmatized populations in Uganda are ready to receive PrEP and that this should encourage Uganda MoH to accelerate the rollout of PrEP and meet the needs of these MARPs. Despite the high interest, PrEP knowledge was low and numerous barriers exist to PrEP use. Based on our findings, the MoH should consider demand creation including public informational campaigns, further HCW training, provision of PrEP near the communities at risk, and promotion of community-based stigma reduction efforts.

## Supporting information

S1 Appendix Survey questionaire—English. (PDF)

S2 Appendix Survey questionaire—Luganda. (PDF)

S1 File Data dictionary. (PDF)

S1 Dataset (CSV)
