# Supplementary material for: Knowledge and barriers of PrEP delivery among diverse groups of potential PrEP users in Central Uganda
Source: PLoS One. 2020 Oct 28;15(10):e0241399. doi: 10.1371/journal.pone.0241399 (PMC7592843; doi:10.1371/journal.pone.0241399)
Supplement: S1 Appendix — (PDF) [file pone.0241399.s001.pdf]

Participant ID: PPU – ☐☐☐

Form Completion Date: ☐☐ ☐☐ ☐☐ (dd mm yy)

☐ *discordant* ☐ *Fisher Fork*
☐ *MSM* ☐ *Female Sex worker*

☐ *pre-training* ☐ *post-training*

PPU Questionnaire, page 1 of 4

1 Date of birth: ☐☐ ☐☐ ☐☐ (*dd* *mm* *yy*)

2 Gender ☐ *female* ☐ *male*

3 What is the participant's marital status?
☐ *single* ☐ *married monogamous* ☐ *married polygamous* ☐ *separated* ☐ *divorced* ☐ *widowed*

4 How many living children does the participant have? ☐☐ *number of children*

5 What is the participant's ethnic group or tribe? ______________________________

6 How many years of school did the participant complete? ☐☐ *number of years*
(***Do not count repeat years***)

7 What is the participant's occupation?
☐ *professional* ☐ *laborer/semi-skilled* ☐ *trade/sales* ☐ *farming/animal raising*
☐ *house wife* ☐ *student* ☐ *other, specify:*______________________

7a. What is the participant's monthly income? (**average over the last 3 months**) ☐☐☐☐☐☐☐

Items 8- 13 are interviewer-administered questions, and should be read aloud directly as written

8 In your lifetime, how do you perceive your risk of getting HIV?
☐ *high risk* ☐ *moderate risk* ☐ *low risk* ☐ *no risk* ☐ *don't know*

8a. Have you ever tested for HIV? ☐ *yes* ☐ *no*

8b. If **yes** in item 8a, how often?
☐ *several times a year* ☐ *once a year* ☐ *less than once a year* ☐ *once ever*

8c In the next one year, what do you think is your risk of acquiring HIV?
☐ *high risk* ☐ *moderate risk* ☐ *low risk* ☐ *no risk* ☐ *don't know*

8d What behaviors place someone at risk for HIV infection? ***mark all that participant mentions***
☐ *sex without a condom* ☐ *multiple partners* ☐ *lack of knowledge of HIV status of sexual partners*
☐ *anal sex* ☐ *concurrent STIs* ☐ *Intravenous Drug Use* ☐ *Other, specify:*______________________

Participant ID: PPU – ☐☐☐ Form Completion Date: ☐☐ (dd) ☐☐ (mm) ☐☐ (yy)

MARPs Questionnaire, page 2 of 4

Items 9 and 11 refer to behavior in the past 30 days. Whenever "sex" or "sexual intercourse" is stated, it includes vaginal and anal sex, but not oral sex. The term "condom" refers to either a male or female condom, lubricant refers to oil used during sex.

9 Do you consider yourself male, female, transgender or other?
☐ *male* ☐ *female* ☐ *transgender* ☐ *other, specify:* ______

9a. **If Male:** Is the participant circumcised? ☐ *yes* ☐ *no*

9b. Do you have sex with Men, women or both? ☐ *men* ☐ *women* ☐ *both*

9c. Do you exchange sex for goods or money? ☐ *yes* ☐ *no* → ***if no go to item 10***

9d. **If yes in item 9c**: Is selling sex your main source of income? ☐ *yes* ☐ *no*

10 In the past 30 days, how many times did you have sexual intercourse? ☐☐ *number of sex acts* → ***if 0 go to 11***

Of those in item 10; 10a. how many were anal sex? ☐☐ *number of anal acts*

10b. How many were vaginal sex? ☐☐ *number of vaginal acts*

10c. How many times was a condom used? ☐☐ *number of times condom used*

10d. How many times was a lubricant used? ☐☐ *number of times lubricant used*

11 In the past 30 days, how many individuals did you have sexual intercourse with? ☐☐ *number of sexual partners* → ***if 0 go to 12***

11a. Of the individuals in 11, how many are of HIV sero-status known to you? ☐☐ *number with known sero-status*

Of the individuals in 11a; 11b. how many are HIV sero-positive? ☐☐ *number with positive status*

11c. how many are HIV sero-negative? ☐☐ *number with negative status*

12 What can be done to reduce chances of getting HIV? ***mark all that participant mentions***
☐ *use condoms* ☐ *limit number of sexual partner* ☐ *STI screening and treatment* ☐ *male circumcision*
☐ *other, specify:* ______

12a. Have you ever heard of PrEP? ☐ *yes* ☐ *no* → ***if no go to 12b***

12b. If **yes** in 12a, describe PrEP? ***mark all that participant mentions***
☐ *Pill to prevent HIV infection* ☐ *Pill taken after potential exposure* ☐ *use of ARV drugs to prevent acquisition of HIV before exposure*
☐ *other, specify:* ______

12c. who are the people that can benefit from PrEP? ***mark all that participant mentions***
☐ *sex workers* ☐ *MSM* ☐ *Sero-discordant couples* ☐ *Fisher Folk* ☐ *other, specify:* ______

12d. How long should someone use PrEP?
☐ *lifelong* ☐ *during periods of risk for HIV acquisition* ☐ *other, specify:* ______

Participant ID: PPU – ☐☐☐ Form Completion Date: ☐☐ dd ☐☐ mm ☐☐ yy

PPU Questionnaire, page 3 of 4

13 ***PrEP is the use of ARV drugs by HIV uninfected persons to prevent acquisition of HIV before exposure*.**

In case PrEP was made available, would you be willing to use PrEP to prevent HIV infection?

☐ *yes* ☐ *no* ☐ *maybe*

if yes, go to item 13b

13a. If no or maybe in item 13, why?

☐ *I need more information* ☐ *not perceived to be at risk* ☐ *happy with other prevention tools like condoms*

☐ *I would want to be seen taking PrEP* ☐ *other, specify:*__________

13b. If yes in item 13, would you be able to take the pill every day? ☐ *yes* ☐ *no* ☐ *maybe*

13c. If **yes** or ***maybe*** in 13b, what do you think would help you remember to take PrEP every day? ***mark all that participant mentions***

☐ *nothing* ☐ *alarm* ☐ *spouse/family members* ☐ *associate it with a daily activity*

☐ *watch/clock* ☐ *pillbox* ☐ *radio* ☐ *other, specify:* __________

13d. What do you think are the possible challenges you may face to get PrEP medication when you need it? ***mark all that participant mentions***

☐ *not wanting to be seen with it/stigma* ☐ *forgetting* ☐ *alcohol use* ☐ *no time to pick it up*

☐ not sure I need it ☐ *other, specify:*__________

14 If you were to accept PrEP, from which facilities would you prefer to get PrEP? ***mark all that apply***

☐ *district hospital* ☐ *health center/dispensary* ☐ *private clinic* ☐ *VCT center* ☐ *MARPs clinic*

☐ *other, specify:*__________

14a why would you prefer to get PrEP from__________? (Insert response from item 14) ***mark all that apply***

☐ *faster service* ☐ *less stigma* ☐ *privacy* ☐ *closer to my home* ☐ *other, specify:*__________

15 People taking PrEP need to test for HIV to make sure they are HIV uninfected. How often would you like to test for HIV?

☐ *every month* ☐ *every 3 months* ☐ *every 6 months* ☐ *once a year* ☐ *other, specify:*__________

Items 16 - 19 deal with feelings you might have if you are taking PrEP and ARV based regimen to prevent HIV infection, We would like to know what you think regardless of whether these things would actually happen. Please tell me if you strongly agree, agree, disagree or strongly disagree with the following four statements.

	**If I acquired HIV…….**	*Strongly agree*	*Agree*	*Disagree*	*Strongly Disagree*
16	I would be treated badly at work or get sacked. OR ☐ *N/A*	☐	☐	☐	☐
17	I would lose friends.	☐	☐	☐	☐
18	My family would disown or neglect me.	☐	☐	☐	☐
19	My community would treat me like an outcast.	☐	☐	☐	☐

Participant ID: **PPU** – ☐☐☐ Form Completion Date: ☐☐ ☐☐ ☐☐ (dd mm yy)

PPU Questionnaire, page 4 of 4

Now I am going to ask you about your alcohol use in the past year. While some of this information may be embarrassing or difficult to remember, please try to give your best answers and be as honest as possible

		yes	*no*
20	During the last year, have you had a feeling of guilt or remorse after drinking?	☐	☐
21	During the last year, has a friend or family member ever told you about things you said or did while you were drinking that you could not remember?	☐	☐
22	During the last year, have you failed to do what was normally expected of you because of drinking?	☐	☐
23	Do you sometimes take a drink in the morning when you first get up?	☐	☐

Item 24 is for sero-discordant participants only

24 Is the HIV- infected partner on ART? ☐ *yes* ☐ *no*

24a. Have you ever disclosed your HIV status to anyone else outside your relationship? ☐ *yes* ☐ *no*

Item 25 is for sex worker participants only

25 What type of sexual partners do you have? ***list all that apply***

☐ *commercial* ☐ *boy friend* ☐ *sponsor/sugar daddy* ☐ *other, specify:* ______________

Item 26 is for MSM participants only

26 Have you disclosed your sexual orientation to any one? ☐ *yes* ☐ *no*

Item 27 is for fish folk participants only

27 How many landing sites does the participant work on? ☐☐ *number of sites*

27a. How many sexual partners does the participant have? ☐☐ *number of partners*

27b.Are all the sexual partners from the same site? ☐ *yes* ☐ *no*
