# Supplementary material for: Knowledge and barriers of PrEP delivery among diverse groups of potential PrEP users in Central Uganda
Source: PLoS One. 2020 Oct 28;15(10):e0241399. doi: 10.1371/journal.pone.0241399 (PMC7592843; doi:10.1371/journal.pone.0241399)
Supplement: S2 Appendix — (PDF) [file pone.0241399.s002.pdf]

Participant ID: PPU – ☐☐☐

☐ *discordant* ☐ *Fisher Fork*
☐ *MSM* ☐ *Female Sex worker*

Form Completion Date: ☐☐ *dd* ☐☐ *mm* ☐☐ *yy*

☐ *pre-training* ☐ *post-training*

1	Date of birth: ☐☐ *dd* ☐☐ *mm* ☐☐ *yy*
2	Gender ☐ *female* ☐ *male*
3	What is the participant's marital status?<br>☐ *single* ☐ *married monogamous* ☐ *married polygamous* ☐ *separated* ☐ *divorced* ☐ *widowed*
4	How many living children does the participant have? ☐☐ *number of children*
5	What is the participant's ethnic group or tribe? ______________________________
6	How many years of school did the participant complete? ☐☐ *number of years*<br>(***Do not count repeat years***)
7	What is the participant's occupation?<br>☐ *professional* ☐ *laborer/semi-skilled* ☐ *trade/sales* ☐ *farming/animal raising*<br>☐ *house wife* ☐ *student* ☐ *other, specify:*______________________<br>7a. What is the participant's monthly income? (**average over the last 3 months**) ☐☐☐☐☐☐☐

Items 8- 27 are interviewer-administered questions and should be read aloud directly as written.

8	***Newankubadde ebintubinoebimu biyinza okubangabikwasa ensonyiobanga bizibu okujjukira, ebintubinoebimu biyinza okubangabikwasa ensonyiobanga bizibu okujjukira, nkusaba ogezeeko okubyanukula mu butuufu bwabyo era ng'oli mwesimbu ngabwekisoboka.***<br>Mubulamu bwo, obulabe byo kukwatibwa akawuka obulaba otya?<br>☐ *obulabe buli wagulu nnyo* ☐ *tebuli awo bwa'maanyi* ☐ *butono* ☐ *tewali bu labe bwonna* ☐ *simanyi*<br>8a. Wali wekebezeseza akawuka ka silimu ☐ *yee* ☐ *nedda*<br>8b. **If yes in item8a**, emirundi emeka?<br>☐ *emirundi mingi mumwaka* ☐ *omurundi gumu mumwaka* ☐ *teguwera gumu mumwaka* ☐ *nakekebeza omulundi gumu kasookedde mbaawo*<br>8c Mumwaka gumu mumaaso, olowooza obulabe bwo kukwatibwa akawuka kasilimu byenkanawa?<br>☐ *obulabe buli wagulu nnyo* ☐ *tebuli awo bwa'maanyi* ☐ *butono* ☐ *tewali bu labe bwonna* ☐ *simanyi*<br>8d Neeyisa ki eteeka omuntu mubulabe bwokukwatibwa akawuka ka silimu? ***mark all that apply***<br>☐ *okwegatta mubibikolwa byomukwano gatokozeseza kondomu* ☐ *okubera nabagalwa abassuka mwomu/ bangi*<br>☐ *obutamanya mbeera yamunno eyakawuka ka silimu* ☐ *okweggatta nga oyita awafulumya obubi*<br>☐ *okubeera nendwadde eziyita mu kweggata ezijja bulikaseera* ☐ *okwekuba eddagala mu musiwa* ☐ *ebirala, biki:*__________________

Participant ID: PPU – ☐☐☐ Form Completion Date: ☐☐ (dd) ☐☐ (mm) ☐☐ (yy)

Obutundu 9, 10 ne 11 bukwata ku neeyisaayo mumwezi oguyise. Okwegatta oba ekikolwa kyokwegattawekyogeddwako, kizingiramu ekyomubukyala nekyo wofulumira obubi, naye tekitegeeza kwegatta nga oyita mukamwa.Ekigambo Kondomu oba akapiira kitegeeza akapiira akomusajja oba akomukazi.Akazigo wekogeddwako kitegeza ekikozesebwa okusereeza nga ekikolwa kyokwegata mumukwano kikolebwa.

9 Gwe weyita musajja, mukazi, mukazi ngate oli musajja oba olina kyeweyita ekirala?

☐ *musajja* ☐ *mukazi* ☐ *mukazi ngate ndi musajja/ musajja ngate ndi mukazi/* ☐ *ebirala, biki:* ____________

9a. **If Male:** Oli mukomole? ☐ *yee* ☐ *nedde*

9b. Wegatta muikolwa ebyomukwano nabasajja, bakazi oba bombi? ☐ *basajja* ☐ *bakazi* ☐ *bombi*

9c. Wegatta nofuna kyoyagala oba sente? ☐ *yee* ☐ *nedda* → ***if no go to item 10***

9d. **If yes in item 9c**: Okwegatta nofuna sente, gewmulimugwo omukulu? ☐ *yee* ☐ *nedda*

10 Munnaku 30 eziyise, emirundi emeka gyewegase mubikolwa ebyomukwano? ☐☐ *emirundi gyo'kwegatta* → ***if 0 go to 11***

Of those in item 10; 10a. Mirundi emeka gyewegase ngo oyita awafulumya obubi? ☐☐ *emirundi gyo'kwegatta*

10b. Mirundi emeka gyewegase ngo oyita mubukyala? ☐☐ *emirundi gyo'kwegatta*

10c. Emirundi emeka gye wakozesa kondomu? ☐☐ *emirundi kondomu lwekozessedwa mukwegatta*

10d. Emirundi emeka gye wakozesa *ekiseza*? ☐☐ *emirundi ekiseza lwekozessedwa mukwegatta*

11 Munnaku 30 eziyise, abantu bameka bewegatta nabo mu bikolwa byo mukwano? ☐☐ *nnamba yabantu be wegasse nabo* → ***if 0 go to 12***

Of the individuals in 11; 11a. Kwabo bewegatta nabo, bameka boomanyi embera zaabwe eza kawuka ka silimu? ☐☐ *nnamba yaabo baamanyi embeera zaabwe azakawuka*

Of the individuals in 11a; 11b. Bameka abalina akawuka kasilimu? ☐☐ *nnamba yaabo abalina akawuka*

11c. Bameka abatalina akawuka kasilimu? ☐☐ *nnamba yaabo abatalina akawuka*

12 Kiki ekiyinza okukolebwa okukendeeza emikisa gy'okukwatibwa akawuka kamukenenya? ***mark all that apply***

☐ *okukozesa akapiiira* ☐ *okukendeeza abantu bewegata nabo* ☐ *okujanjaba endwadde ze kyama* ☐ *okukomolebwa okwekisawo*

☐ *ebirala, biki:* ____________

12a.Wali owuliddeko kunkola ya PrEP/eddagala erimiribwa olwo olwo? ? ☐ *yee* ☐ *nedda*

12b. If **yes** in 12a, Nnyonyola PrEP kyeki? ***mark all that participant mentions*** → ***if no go to 12e***

☐ *ekerenda eriziyiza okukwatibwa akawuka ka silimu* ☐ *ekerenda erimiribwa nga olowooza wegasse noyo alina akawuka* ☐ *okukozesa ARV okuziyiza akawuka ka silimu nga tonaba kukafuna*

☐ *ebirala, biki:* ____________

12c.Ani yakubulira ku nkola ya PrEP? ***mark all that participant mentions***

☐ *abasawo* ☐ *kukikutu gyempuliganya* ☐ *mi kwano* ☐ *ebirala, biki:* ____________

12d. Abantu ki bayinza okufunamu nga ba kozeseza PrEP? ***mark all that participant mentions***

☐ *abantu abegatta olwa sente* ☐ *abasajja abegatta nebasajja banabwe* ☐ *Sero-discordant couples* ☐ *abavubi* ☐ *ebirala,:* ____________

12e. Banga lyenkana wa omuntu lye yandimaze nga akozesa PrEP?

☐ *lebanga lyonna eryobulamu* ☐ *mubisera omuntu nga ali mukatyabaga ka ku kwatibwa akawuka* ☐ *ebirala:* ____________

Participant ID: PPU – ☐☐☐ Form Completion Date: ☐☐ (dd) ☐☐ (mm) ☐☐ (yy)

13 **PrEP kwekukozesa eddagala eriweweza akawuka ka silimu/ARVs ngalikozesedwa abatalina kawuka okuzizaokukwatibwa nga tebanaba kubera mumbera eyinza okukabasiiga**.

Ssinga PrEP abaawo, wandyagadde okumukozesa okuziyiza okukwatibwa akawuka ka silimu?

☐ *yee* → ***if yes, go to item 13b*** ☐ *nedda* ☐ *obolyawo*

13a. **If no or maybe in item 13**, Lwaki?

☐ *netaaga obubaka obusingawo* ☐ *siri mu katyabaga ka kukwatibwa silimu* ☐ *ndi musanyufu nenkola endala eyokuziyiza okukwtibwa akawuka ka silimu nga kondomu*

☐ *sandyagadde kundaba nga mmira eddagala lya PrEP* ☐ *ebirala, biki:* ____________

13b. **If yes in item 13**, Wandyagadde okumira eddagala eryo buli lunaku? ☐ *yee* ☐ *nedda* ☐ *obolyawo*
→ ***if no go to 13d***

13c. **If yes or *maybe* in 13b**, Kiiki kyolowoza ekiyinza okukuyamba okujjukira okumira eddagala lya PrEP? buli lunnaku? ***mark all that participant mentions***

☐ *tewali* ☐ *akadde* ☐ *omwagalwa wange* ☐ *mbikwataganya nebikolwa/emirimu gyange egya buli lunaku*

☐ *esaawa* ☐ *akakebe mwentereka eddagala* ☐ *radio* ☐ *TV* ☐ *ebirala, biki:*____________

13d. Olowooza bik ebiyinza okukuwa obuzibu mukufuna eddagala lya PrEP singa oba olyetaze?
mark all that participant mentions

☐ *obutagala kundaba nalyo* ☐ *okwerabira* ☐ *okunnywa omwenge* ☐ *obutafuna budde bwakulinona*

☐ *nga sekakasa nti ndyetaaga* ☐ *ebirala, biki:* ____________

14 Ssinga oba wakukkiriza PrEP, bifo ki byewandyagadde okulifunira?

☐ *kumalwaliro ga disitulikiti* ☐ *kumalwaliro ga HC/dispensale* ☐ *kumalwaliro go bwananyini* ☐ *VCT center* ☐ *MARPS clinic*

☐ *ebirala, biki:* ____________

14a Lwa wandyagadde okufuna PrEP okuva mu ______? (*Insert response from item 14*) ***mark all that apply***

☐ *banguya* ☐ *tewali kusosolwa lwembera gyolimu* ☐ *waliwo okukuma ebyama okumala* ☐ *kumpi ne awaka* ☐ *ebirala:*____________

15 abantu abamira PrEP beetaga okukeberebwa akawuka ka silimu okukasa nti tebakalina. Emirundi emeka gyewandyagadde okukeberebwa akawuka ka silimu?

☐ *buli mwezi* ☐ *buli luvanyuma lwa myezi essatu* ☐ *buli luvanyuma lwa myezi mukaaga* ☐ *mulundigumu mu mwaka* ☐ *ebirala:* ____________

Ebibuuzo namba 16-19 bikwata kungeri gyoyinza okuyisibwaamu nga omira eddagala lya PrEP ne ARVs okuziyiza akawuka akaleta mukenenya, Twagalaokumanya ky'olowooza nga tetusinziira kukuba nti ebintu ebyo binaabeerawo oba nedda. Mbulira oba nga okiririza ddala, okiriza, tokiriza oba tokiririza ddala, kubino ebina ebyogeddwaako wamanga;

	**Singa mba nga mira PrEP oba ARVs…….**	*Nzikiririza ddala*	*Nzikiriza*	*Ssikiriza*	*Ssikiririza Ddala*
16	Njakuyisibwa bubi kumulimu oba bajjakungoba.Oba ☐ *Tekisanira*	☐	☐	☐	☐
17	Nja kuvibwaako emikwako	☐	☐	☐	☐
18	Famile yange ejjakunvaamu	☐	☐	☐	☐
19	Abomukitundu kyange bajja kumpisa nga ekitagasa.	☐	☐	☐	☐

Participant ID: PPU – ☐☐☐ Form Completion Date: ☐☐ dd ☐☐ mm ☐☐ yy

PPU Questionnaire, page 4 of 4

Obutundu 20 - 23 bukwata ku ngeri gy'okozesezza omwenge mu bbanga ery'omwaka ogumu oguyise.

		yee	nedda
20	Mu bbanga ery'omwaka ogumu oguyise, wawulirako muli obuswavu oba okwejjusa oluvannyuma lw'okunywa omwenge ?	☐	☐
21	Mubbanga ery'omwakaogumu oguyise, waliwo mukwano gwoo ba owolugandalwo eyali akugambyeko ebintu byewayogera obabyewakolang'onywedde omwenge kyokkangaggwe tobijjukira?	☐	☐
22	Mubbangaery'omakaogumu oguyise, walemererwako okukola ekintu kyonna kyewali osuubirwa okukola olw'okunywa omwenge?	☐	☐
23	Waliwo oluusi lw'osookaokunywa ekintu kyona kumakya nga wakagolokoka?	☐	☐

Item 24 is for sero-discordant participants only

24	Omwagalwa wo alina akawuka kasilimu ali kuddagala?	☐ *yee*	☐ *nedda*
	24a. Wali obuliddeko omuntu yena kumbeera yo eya kawuka ka silimu nga ogyeko Omwagalwa wo?	☐ *yee*	☐ *nedda*

Item 25 is for sex worker participants only

25 Bantu bakika ki bewegatta nabo bolina? ***list all that apply***

☐ *abo aba ssente* ☐ *bakwano bange* ☐ *abampa byenjagala oba abasajja abakulu* ☐ *abala bani:* ____________________

Item 26 is for MSM participants only

26	Wali obuliddeko omuntu yenna enegatta yo eno?	☐ *yee*	☐ *nedda*

Item 27 is for fish folk participants only

27	Emyalo emeka gyo kolero ko?	☐☐ *namba ye myalo*
	27a. Olina bewegatta nabo mumukwano bameka?	☐☐ *namba yabo be negatta nabo*
	27b. Bonna bewegatta nabo bava kumwalo gwegumuko?	☐ *yee* ☐ *nedda*
