# Supplementary material for: Knowledge and barriers of PrEP delivery among diverse groups of potential PrEP users in Central Uganda
Source: PLoS One. 2020 Oct 28;15(10):e0241399. doi: 10.1371/journal.pone.0241399 (PMC7592843; doi:10.1371/journal.pone.0241399)
Supplement: S1 File — (PDF) [file pone.0241399.s003.pdf]

Data dictionary_Knowledge and Barriers of PrEP Delivery_in Central Uganda

No	Variable_name	Generic Name	Type	Description	Legal Range	Format / Choices / Display width
1	PTID_Req	PTID	str	Participant ID	001-999	Format = nnn
2	Form_year	FormYear	dt	Form completion Year	2017-2018	Format = yyyy
3	SimpleChoice	ParticipantType	int	Type of participant:	1,2,3,4	1 = serodiscordant couples<br>2 = fisher folk<br>3 = men who have sex with men<br>4 = female sex workers
4	SimpleChoice	SurveyType	int	Type of survey:	1,2	1 = pre-training<br>2 = post-training
5	SimpleChoice	Recruitment source	int	Recruitment source	1,2,3	1 = urban<br>2 = peri-urban<br>3 = rural
6	birth_year	BirthYear	dt	Year of Birth	1950 – 2000	Format = yyyy
7	SimpleChoice	Sex	int	Sex of Participant	1,2	1 = female<br>2 = male
8	SimpleChoice	MaritalStatus	int	Participant's marital status	1,2,3,4,5,6	1 = single<br>2 = married monogamous<br>3 = married polygamous<br>4 = separated<br>5 = divorced<br>6 = widowed
9	SimpleNumber	YrsSchool	int	How many years of school did the participant complete?	0-25	Format = nn

10	SimpleChoice	Occupation	int	What is the participant's occupation?	1,2,3,4,5,6,7	1 = professional<br>2 = laborer/semi-skilled<br>3 = trade/sales<br>4 = farming/animal raising<br>5 = house wife<br>6 = student<br>7 = other specify
11	SimpleNumber	MonthIncome	int	What is the participant's monthly income (UGX)?		Format = nnnnnnnn
12	SimpleChoice	HIVLifetimeRisk	int	In your lifetime, how do you perceive your risk of getting HIV?	1,2,3,4,5	1 = high risk<br>2 = moderate risk<br>3 = low risk<br>4 = no risk<br>5 = don't know
13	SimpleChoice	Gender	int	Do you consider yourself male, female, transgender or other?	1,2,3	1 = female<br>2 = male<br>3 = transgender
14	SimpleChoice	HeardPrEP	int	Have you ever heard of PrEP?	1,2	1 = yes<br>2 = no
15	SimpleCheck	DP_PillHIVtreat	ck	Describe PrEP: pill to treat HIV infection		0 = 0<br>1 = 1
16	SimpleCheck	DP_PillAfterHIVExposure	ck	Describe PrEP: pill taken after potential exposure to HIV		0 = 0<br>1 = 1
17	SimpleCheck	DP_ARVPreventHIV	ck	Describe PrEP: use of ARV drugs to prevent acquisition of HIV before		0 = 0<br>1 = 1

Data dictionary_Knowledge and Barriers of PrEP Delivery_in Central Uganda

				exposure		
18	SimpleCheck	DP_others	ck	Describe PrEP: others		0 = 0<br>1 = 1
19	Comments	DP_specify	str	Specify other descriptions of PrEP		Display width = 200
20	SimpleCheck	PrEPInfo_HCW	ck	Who told you about PrEP: Health Care Worker		0 = 0<br>1 = 1
21	SimpleCheck	PrEPInfo_Media	ck	Who told you about PrEP: Media		0 = 0<br>1 = 1
22	SimpleCheck	PrEPInfo_Friend	ck	Who told you about PrEP: Friend		0 = 0<br>1 = 1
23	SimpleCheck	PrEPInfo_Other	ck	Who told you about PrEP: Other		0 = 0<br>1 = 1
24	Comments	PrEPInfo_specify	str	Specify other PrEP information sources		Display width = 200
25	SimpleCheck	PrEPBenefit_sexworkers	ck	PrEP beneficiaries: sex workers		0 = 0<br>1 = 1
26	SimpleCheck	PrEPBenefit_MSM	ck	PrEP beneficiaries: men who have sex with men		0 = 0<br>1 = 1
27	SimpleCheck	PrEPBenefit_SDC	ck	PrEP beneficiaries: serodiscordant couples		0 = 0<br>1 = 1
28	SimpleCheck	PrEPBenefit_FF	ck	PrEP beneficiaries: fisher folk		0 = 0<br>1 = 1
29	SimpleCheck	PrEPBenefit_Alcoholusers	ck	PrEP beneficiaries: Alcohol users		0 = 0<br>1 = 1
30	SimpleCheck	PrEPBenefit_Bfeedmothers	ck	PrEP beneficiaries: Breastfeeding Mothers		0 = 0<br>1 = 1

Data dictionary_Knowledge and Barriers of PrEP Delivery_in Central Uganda

31	SimpleCheck	PrEPBenefit_HIV+persons	ck	PrEP beneficiaries: HV positive persons		0 = 0<br>1 = 1
32	SimpleCheck	PrEPBenefit_NonHIVperons	ck	PrEP beneficiaries: HIV negative persons		0 = 0<br>1 = 1
33	SimpleCheck	PrEPBenefit_MAPRS	ck	PrEP beneficiaries: Most At Risk Populations		0 = 0<br>1 = 1
34	SimpleCheck	PrEPBenefit_Raped	ck	PrEP beneficiaries: Raped Persons		0 = 0<br>1 = 1
35	SimpleCheck	PrEPBenefit_Youth	ck	PrEP beneficiaries: youth		0 = 0<br>1 = 1
36	SimpleCheck	PrEPBenefit_Dontknow	ck	PrEP beneficiaries: Don't Know		0 = 0<br>1 = 1
37	SimpleChoice	PrEPDuration	int	How long should someone use PrEP?	1,2,3,4,5,6,7	1 = lifelong<br>2 = During periods of risk for HIV acquisition<br>3 = 1 month or less<br>4 = 3-6 months<br>5 = 1 year<br>6 = 2 years<br>7 = Dont know
38	SimpleChoice	PrEPWillingness	int	In case PrEP was made available, would you be willing to use it to prevent HIV infection?	1,2,3	1 = yes<br>2 = no<br>3 = maybe
39	SimpleCheck	NonPrEP_MoreInfo	ck	Reason for no PrEP use: Need more Information		0 = 0<br>1 = 1

40	SimpleCheck	NonPrEP_NoRisk	ck	Reason for no PrEP use: Not perceived to be at risk		0 = 0<br>1 = 1
41	SimpleCheck	NonPrEP_Happyother	ck	Reason for no PrEP use: Happy with other prevention tools like condoms		0 = 0<br>1 = 1
42	SimpleCheck	NonPrEP_Notseen	ck	Reason for no PrEP use: I would not want to be seen taking PrEP		0 = 0<br>1 = 1
43	SimpleCheck	NonPrEP_SideEffects	ck	Reason for no PrEP use: Side Effects		0 = 0<br>1 = 1
44	SimpleCheck	NonPrEP_PillBurden	ck	Reason for no PrEP use: Pill Burden		0 = 0<br>1 = 1
45	SimpleCheck	Challenge_stigma	ck	Possible challenges to get PrEP: Stigma		0 = 0<br>1 = 1
46	SimpleCheck	Challenge_Forgetting	ck	Possible challenges to get PrEP: Forgetting		0 = 0<br>1 = 1
47	SimpleCheck	Challenge_Alcholuse	ck	Possible challenges to get PrEP: Alcohol use		0 = 0<br>1 = 1
48	SimpleCheck	Challenge_BusySchedule	ck	Possible challenges to get PrEP: Busy Schedule		0 = 0<br>1 = 1
49	SimpleCheck	Challenge_Notneed	ck	Possible challenges to get PrEP: Not sure I need it		0 = 0<br>1 = 1
50	SimpleCheck	Challenge_Transport	ck	Possible challenges to get PrEP: Transport		0 = 0<br>1 = 1
51	SimpleCheck	Challenge_Accessibility	ck	Possible challenges to get PrEP: Accessibility		0 = 0<br>1 = 1
52	SimpleCheck	Challenge_DrugCost	ck	Possible challenges to get PrEP: Drug Cost		0 = 0<br>1 = 1

53	SimpleCheck	Challenge_DrugStockout	ck	Possible challenges to get PrEP: Drug Stockout		0 = 0<br>1 = 1
54	SimpleCheck	Challenge_HCWAttitude	ck	Possible challenges to get PrEP: Health Care Worker Attitude		0 = 0<br>1 = 1
55	SimpleCheck	Challenge_KnowledgeGap	ck	Possible challenges to get PrEP: Knowledge Gap		0 = 0<br>1 = 1
56	SimpleCheck	Challenge_PillBurden	ck	Possible challenges to get PrEP: Pill Burden		0 = 0<br>1 = 1
57	SimpleCheck	Challenge_SideEffects	ck	Possible challenges to get PrEP: Side Effects		0 = 0<br>1 = 1
58	SimpleCheck	PreferredFacility_DistrictHospital	ck	From which facilities would you prefer to get PrEP: District Hospital		0 = 0<br>1 = 1
59	SimpleCheck	PreferredFacility_HealthCenter	ck	From which facilities would you prefer to get PrEP: Health Center		0 = 0<br>1 = 1
60	SimpleCheck	PreferredFacility_PrivateClinic	ck	From which facilities would you prefer to get PrEP: Private Clinic		0 = 0<br>1 = 1
61	SimpleCheck	PreferredFacility_VCT	ck	From which facilities would you prefer to get PrEP: VCT Center		0 = 0<br>1 = 1
62	SimpleCheck	PreferredFacility_MARPClinic	ck	From which facilities would you prefer to get PrEP: MARP Clinic		0 = 0<br>1 = 1
63	SimpleCheck	PreferredFacility_Other	ck	From which facilities would you prefer to get PrEP: Other		0 = 0<br>1 = 1
64	SimpleCheck	ReasonPrefer_Fasterservice	ck	Reason why prefer stated facilities: Faster service		0 = 0<br>1 = 1
65	SimpleCheck	ReasonPrefer_LessStigma	ck	Reason why prefer stated facilities: Less Stigma		0 = 0<br>1 = 1

Data dictionary_Knowledge and Barriers of PrEP Delivery_in Central Uganda

66	SimpleCheck	ReasonPrefer_Privacy	ck	Reason why prefer stated facilities: Privacy		0 = 0<br>1 = 1
67	SimpleCheck	ReasonPrefer_CloserHome	ck	Reason why prefer stated facilities: Closer to my Home		0 = 0<br>1 = 1
68	SimpleCheck	ReasonPrefer_Accessibility	ck	Reason why prefer stated facilities: Accessibility		0 = 0<br>1 = 1
69	SimpleCheck	ReasonPrefer_Availability	ck	Reason why prefer stated facilities: Availability		0 = 0<br>1 = 1
70	SimpleCheck	ReasonPrefer_Convenience	ck	Reason why prefer stated facilities: Convenience		0 = 0<br>1 = 1
71	SimpleCheck	ReasonPrefer_FreeServices	ck	Reason why prefer stated facilities: Free Services		0 = 0<br>1 = 1
72	SimpleCheck	ReasonPrefer_GoodHCW	ck	Reason why prefer stated facilities: Good attitude and experience of Health Care Workers		0 = 0<br>1 = 1
73	SimpleCheck	ReasonPrefer_Trust	ck	Reason why prefer stated facilities: Trust and Reliability		0 = 0<br>1 = 1

Key: int – Integer, dt – Date, ck – Check box, str - String
